# The Evolving Landscape of Medical Device Regulation in East, Central, and Southern Africa

**DOI:** 10.9745/GHSP-D-20-00578

**Published:** 2021-03-31

**Authors:** Sarah Hubner, Caroline Maloney, Sarah Dunn Phillips, Pratik Doshi, Julius Mugaga, Robert Tamale Ssekitoleko, Jenna L. Mueller, Tamara N. Fitzgerald

**Affiliations:** a Trinity College of Arts and Sciences, Duke University, Durham, NC, USA.; b Margolis Center for Health Policy, Duke University, Durham, NC, USA.; c School of Medicine, Duke University, Durham, NC, USA.; d Duke Global Health Institute, Durham, NC, USA.; e Biomedical Engineering Unit, Makerere University, Kampala, Uganda.; f Clark School of Engineering, University of Maryland, College Park, MD, USA.

## Abstract

Effective regulatory frameworks, harmonized to international standards, are critical to expanding access to quality medical devices in low- and middle-income countries. This review provides a summary of the state of medical device regulation in the 14 member countries of the College of Surgeons of East, Central, and Southern Africa (COSECSA) and South Africa. Countries were categorized according to level of regulatory establishment, which was found to be positively correlated to gross domestic product (GDP; r_s_=0.90) and years of freedom from colonization (r_s_=0.60), and less positively correlated to GDP per capita (r_s_=0.40). Although most countries mandate medical device regulation in national legislation, few employ all the guidelines set forth by the World Health Organization. A streamlined regulatory process across African nations would simplify this process for innovators seeking to bring medical devices to the African market, thereby increasing patient access to safe medical devices.

## Abstract (precis)

Most existing medical devices were not built for the challenges often present in many African countries. Regulatory systems for medical devices are essential to ensuring device safety and efficacy. Yet, currently, most African countries do not have a well-defined regulatory process. This discourages both innovators within Africa and companies outside of Africa from developing quality medical devices suitable for these challenges.

## Teaser

**Key Findings:**

- Although 11 of 14 member countries of the College of Surgeons of East, Central, and Southern Africa have legislation mandating the regulation of medical devices, only half are currently developing medical device regulatory processes and half do not have a formal process.
- A country's gross domestic product had a strong correlation to level of medical device regulation.
- The number of years that had elapsed from the country's independence to the present had a strong correlation to the status of the country's regulatory processes.

**Key Implications:**

- To expand regulatory capacity and minimize resource expenditure when developing or creating new medical device regulations, policy makers should facilitate adopting or amending existing harmonized regulations.
- Ministries of health should prioritize local capacity building in the form of well-trained personnel, tools, and facilities to improve regulatory standards.
- Medical technology companies with a charitable division should consider investment in capacity building and innovation to harmonize regulatory standards across African countries.

## INTRODUCTION

Medical devices are essential to the diagnosis and treatment of many diseases, particularly within surgical specialties, radiology, and critical care.^1^ A medical device is any instrument, apparatus, machine, appliance, implant, reagent for in vitro use, software, material, or related article used for a specific medical purpose.^2^ Most existing medical devices were built for the demands and resources available in high-income countries and are not adapted to the challenges often present in many countries in Africa.^3^ Therefore, there is an urgent need to develop medical devices that are specifically designed to address these challenges to improve African patients' access to medical care.^4^ The medical device regulatory processes in many African countries are not well-defined, and countries may rely on clearance from the European Medicines Agency^5^ or the U.S. Food and Drug Administration (FDA).^6^ Although these regulatory processes are stringent with excellent safety standards,^7^ these processes are expensive and may be prohibitive to nonprofit organizations or local device developers in Africa. In addition, the regulatory processes of high-income countries are not designed to meet the needs and safety issues present in Africa. Further, it can be challenging to obtain regulatory approval or clearance in multiple African countries since regulatory processes vary or can be challenging to navigate.^8^

Well-established regulatory systems for medical devices are essential to ensuring device safety and efficacy.^9^ In 1993, the Global Harmonization Task Force (GHTF), now known as the International Medical Device Regulators Forum (IMDRF), was founded in association with multiple national regulatory authorities. The IMDRF encourages convergence of regulatory standards for medical devices and facilitates information access for countries in the development phase of their regulatory process.^10^ Despite these efforts, very few African countries have established regulatory systems. A 2017 World Health Organization (WHO) report found that 40% of countries in the WHO-defined African region have no regulations for medical devices, 32% have some regulations, and the remaining 28% have no available data. In contrast, medical device regulation is present in 58% of all WHO member countries.^11^ This gap in medical device regulation between the African region and the global average is important to address as it may translate to lower quality medical devices and limited access to health care technology for patients.

The importance of medical device regulation is magnified by the prevalence and economic cost of substandard medicines and medical devices. According to the WHO, in 2017, the approximate failure rate of substandard and falsified medical products in low- and middle-income countries was 10.5%, which translates to an economic loss of around $30.5 billion in medical expenditures. Strong medical device regulation is therefore an important, needed step toward achieving higher-quality and more affordable medical care for countries already working within tight economic constraints.^12^

Underdeveloped regulatory processes present challenges for businesses and manufacturers of new medical devices interested in entering the African market,^13^ as regulatory processes are country-dependent but generally modeled after the European Union and the Medical Device Directive.^10^ As a result, introducing a new medical device in the African region requires evaluating local laws and regulations on a country by country basis.

Previous evaluations of regulatory work have been published.^8^^,^^14^^–^^17^ We provide an updated review with a focus on medical device regulation in the 14 member countries of the College of Surgeons of East, Central, and Southern Africa (COSECSA).^18^ COSECSA is the largest surgical training institution in sub-Saharan Africa, with a diverse international surgical membership who commonly use a wide range of medical devices. This summary is essential to understanding the state of medical device regulations in this region of Africa, examining how regulatory systems could be further developed and harmonized, and developing best approaches for increasing access to new medical devices in COSECSA countries and surrounding regions.

This review is essential to understanding the state of medical device regulations in this region of Africa, examining how regulatory systems could be further developed and harmonized, and developing best approaches for increasing access to new medical devices.

## METHODS

### Search Strategy and Selection Criteria

We completed a literature review to understand the status of medical device regulation in COSECSA countries and South Africa. The following databases were searched for peer-reviewed journal articles up to December of 2019: SCOPUS, PubMed, and Google Scholar. Search terms included “medical device regulation,” “device regulation,” “Africa,” and “sub-Saharan Africa,” as well as the individual countries under consideration. Literature detailing regulation of medical devices outside of the African countries of interest were excluded from this review. Literature that discussed only the regulation of medicines and pharmaceuticals and not medical devices was also excluded. The relevant literature was agreed upon by 2 reviewers and examined. Additional sources were identified within the reference lists of literature compiled during this initial search. A Google search was conducted for non-peer-reviewed gray literature, including government legislation and reports by both governmental and nongovernmental organizations. This search provided access to country-specific information, legislation from national regulatory authority websites, and reports from nongovernmental organizations and the United Nations.

Key information was extracted from relevant literature and organized by country. Data included: national regulatory authorities or regulatory bodies; regulatory legal framework; medical device definition; device classification system; essential principles and standards; conformity assessment; registration and listing requirements; import controls; and postmarket controls. These key areas were adapted from WHO guidelines.^11^

A classification scheme was developed to categorize the level of medical device regulation. Level 1 was designated for countries with the most well-established regulatory processes. These may closely resemble those of the FDA or European Medicines Agency in both complexity and level of establishment. Level 2 was designated for countries with developing regulatory processes where such processes are not yet well-established or implemented. Lastly, level 3 was designated for countries with no defined regulatory approval process for medical devices. This included countries that have legislation mandating the regulation of medical devices but have no defined system for pursuing implementation. It also included countries that use informal systems of regulation or regulate medical devices according to the same policies that govern the import of all commercial goods.

Country regulatory levels were correlated to gross domestic product (GDP), GDP per capita,^19^ and years since freedom from colonization by calculating the Spearman correlation coefficient in Microsoft Excel (Office 365 version 16.41). A correlation coefficient of 0 indicates no correlation, while a coefficient of 1 indicates perfect correlation between variables. Ethical review by an institutional review board was not sought as all information was accessed from publicly available sources.

## RESULTS

The literature search returned 6,138 articles, of which 11 were determined to be relevant and were reviewed. Additional sources included 10 government websites, 16 nongovernmental organization websites, and 4 publicly available, non-peer-reviewed websites.

### GDP, Colonization, and Regulatory Processes

**FIGURE 1:**
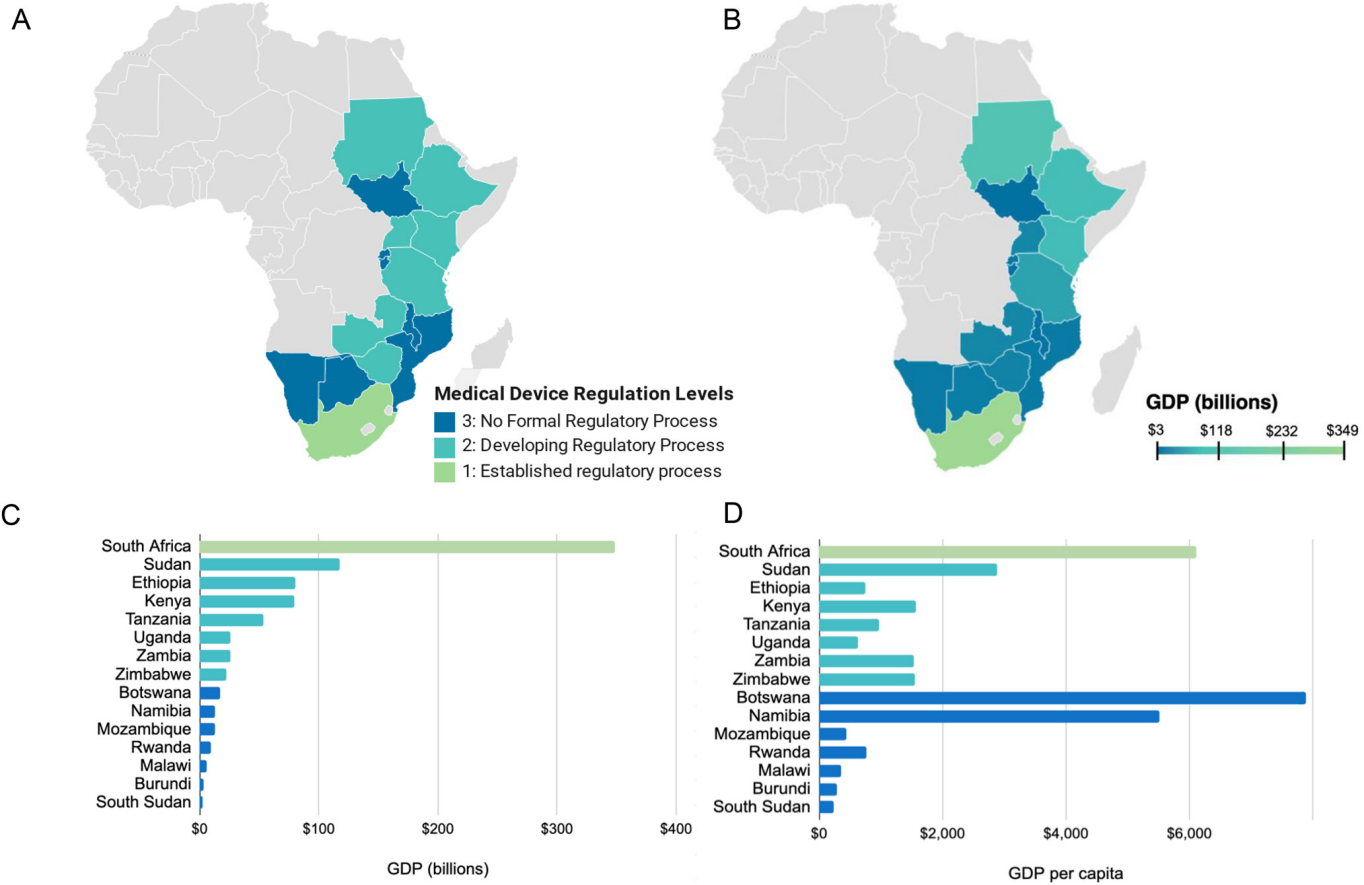
(A) Map of Africa showing the levels of medical device regulation in selected countries. (B) Map of Africa showing the GDP in selected countries in 2020. (C) The level of medical device regulation is correlated to gross domestic product (Spearman correlation coefficient of 0.90). (D) The level of medical device regulation is not significantly correlated to the 2020 gross domestic product per capita (Spearman correlation coefficient of 0.40).

All COSECSA countries and South Africa were evaluated to determine their respective levels of medical device regulation (Figure 1A). South Africa, though not a COSECSA member country, was included in analysis as a point of comparison. Half of all COSECSA countries (n=7, 50%) are currently developing regulatory processes for medical devices (Level 2) while the remaining half (n=7, 50%) do not have a formal regulatory process in place for medical devices (Level 3). South Africa has an established, formal regulatory process for medical devices that includes all essential regulatory components as recommended by the WHO (Level 1).

Levels of medical device regulation were examined with respect to GDP (Figure 1B, C) and GDP per capita (Figure 1D) as these metrics are descriptive of the size of the economy and income per person. GDP was found to have a strong positive association with the level of medical device regulation, yielding a Spearman correlation coefficient of 0.90. South Africa, with the highest GDP of $349 billion,^19^ has the greatest establishment of medical device regulation. All countries with a GDP between $20 and $120 billion fell under Level 2. All countries with a GDP lower than $20 billion fell under Level 3.

Interestingly, the same trend was not as prominent for GDP per capita, where the Spearman correlation coefficient was 0.40, indicating a weak association. Botswana and Namibia, with the highest and third highest GDP per capita respectively, both fall under Level 3. South Africa has the second highest GDP per capita and falls under Level 1. In summation, GDP has a strong correlation with medical device regulation while GDP per capita shows a less clear association.

GDP has a strong correlation with medical device regulation, but GDP per capita shows a less clear association.

**FIGURE 2:**
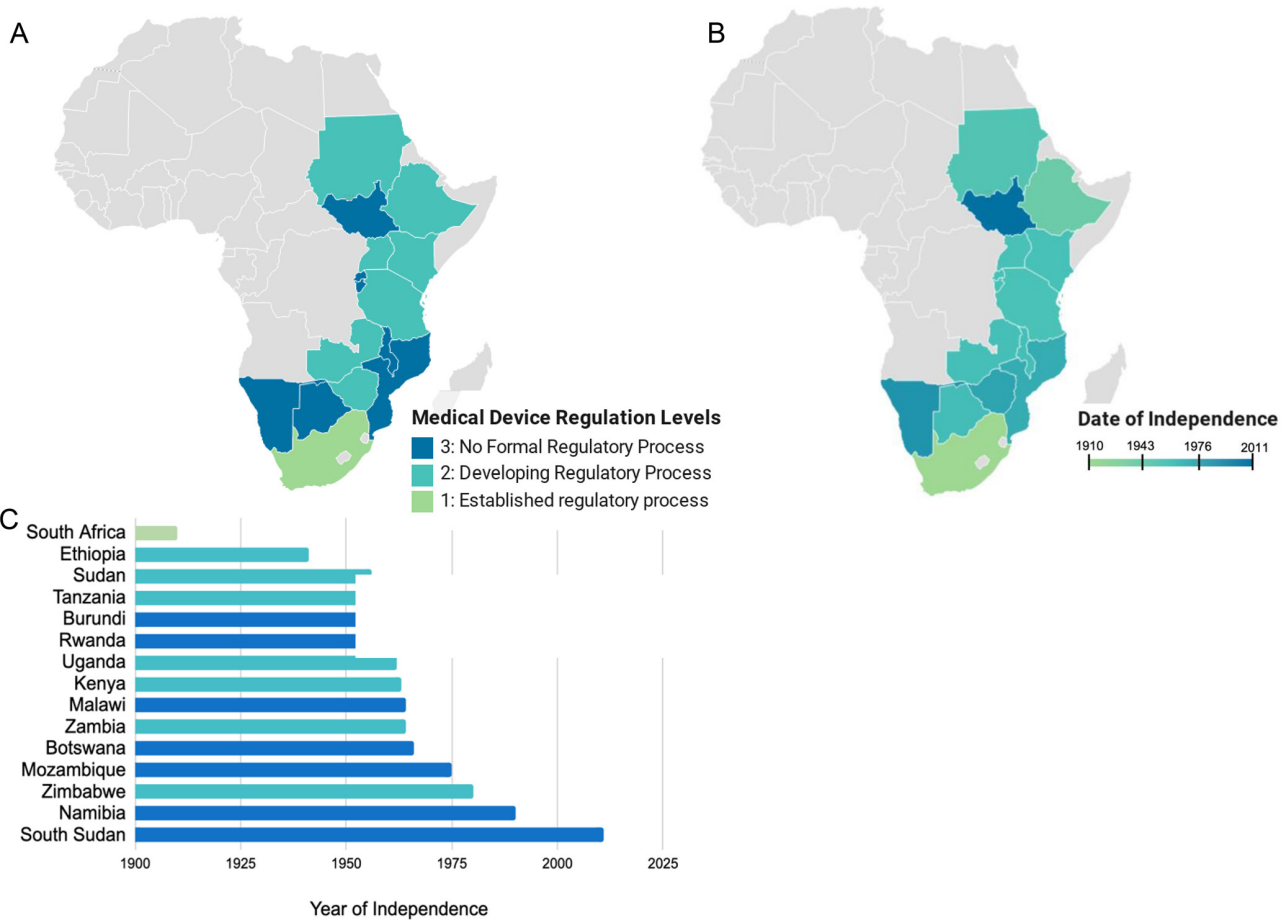
(A) Map of Africa showing the levels of medical device regulation in selected countries. (B) Map of Africa showing dates of country independence. (C) The level of medical device regulation is correlated to the year of independence (Spearman correlation coefficient of 0.60).

Due to a history of colonization in sub-Saharan Africa, and its negative sequelae,^20^ we examined years of country independence and compared it to the status of medical device regulation (Figure 2). Years of independence was defined as the number of years elapsed from the date of the country's independence to the present. In general, the longer a country has existed as an independent state, the more advanced the regulatory process. The correlation coefficient between regulatory status and years of independence was 0.60, indicating a strong correlation.

### Critical Components of the Regulatory Process

An overview of the regulatory processes of the COSECSA countries and South Africa is presented in the Table. This includes information regarding the existence of certain premarket controls, placing on the market, and postmarket controls recommended within the *2017 WHO Global Model Regulatory Framework for Medical Devices*.^11^

**TABLE.** Existence of Critical Components of the Regulatory Process for Medical Devices in COSECSA Countries and South Africa

	Regulatory Complexity 1–3	Regulatory Body	Legal Framework	Medical Device Defined	Risk-based Classification System	Essential Principles	Conformity Assessment	Registration Required	Import Controls	Post-Market Controls
South Africa	1	South African Health Products Regulatory Authority	**✓**	**✓**	A–D	**✓**	**✓**	**✓**	**✓**	**✓**
Sudan	2	National Medicine and Poisons Board	**✓**	**✓**	A–D	**✓**	**✓**	**✓**	X	**✓**
Ethiopia	2	Food, Medicine and Healthcare Administration and Control Authority	**✓**	**✓**	I–IV	**✓**	X	**✓**	X	**✓**
Kenya	2	The Pharmacy and Poisons Board	**✓**	**✓**	A–D	**✓**	**✓**	**✓**	**✓**	**✓**
Tanzania	2	Tanzania Food and Drugs Authority	**✓**	**✓**	A–D	**✓**	**✓**	**✓**	**✓**	**✓**
Uganda	2	National Drug Authority	**✓**	**✓**	X	X	**✓**	**✓**	**✓**	**✓**
Zambia	2	Zambia Medicines Regulatory Authority	**✓**	**✓**	X	X	X	**✓**	**✓**	**✓**
Zimbabwe	3	Medical Devices Unit, Medicines Control Authority	**✓**	X	X	X	**✓** ^a^	**✓** ^a^	**✓** ^a^	**✓** ^a^
Botswana	3	Botswana Medicines Regulatory Authority	**✓**	**✓**	X	X	X	X	X	X
Namibia	3	Medicines Regulatory Council	**✓**	**✓**	X	X	X	X	X	X
Mozambique	3	None	X	X	X	X	X	X	X	X
Rwanda	3	Rwanda Food and Drug Administration	**✓**	**✓**	**✓**	X	X	**✓**	**✓**	**✓**
Malawi	3	Pharmacy, Medicines & Poisons Board	X	X	X	X	X	X	X	X
Burundi	3	Directorate of Pharmacies, Medicines and Laboratories	X	X	X	X	X	X	X	X
South Sudan	3	Drug and Food Control Authority	**✓**	**✓**	X	X	X	X	X	X

Abbreviation: COSECSA, College of Surgeons of East, Central and Southern Africa.

a Only for gloves and condoms.

#### 1. Legal Framework

The establishment of medical device regulation must have a sound legal basis. Although the legal foundation can vary, the WHO recommends legislation to define the scope of regulation. This should include a formalized definition of a medical device, one that is ideally harmonized to the WHO definition. It should also require that only medical devices that are safe, of acceptable quality, and perform as intended can be marketed. Additionally, it should mandate the formation of a regulatory authority and establish the responsibilities and enforcement capabilities of that agency.^11^

All COSECSA member countries and South Africa with the exception of Burundi,^21^ Malawi,^22^ and Mozambique^23^ have legislation mandating the regulation of medical devices. The specificity of this legislation varies with the level of regulatory establishment. South Africa, for example, regulates medical devices according to 3 distinct pieces of legislation and guidelines most closely resembling those of the IMDRF founding members.^24^ Level 3 countries including Botswana,^25^ Burundi,^21^ Rwanda,^26^ South Sudan,^27^ and Zimbabwe^28^ use a legal framework for the regulation of medical devices but in a more limited capacity. This legislation is restricted to the mention of medical devices and definitions of medical devices in legislative acts establishing national medicines regulatory authorities. It does not assign specific responsibilities or guidelines for regulation.

The South Sudanese *Drug and Food Control Authority Act, 2012*, for example, states^29^:

*The purpose of this act is to provide for the establishment of an independent Drug and Food Control Authority in South Sudan and to provide an appropriate and effective independent regulatory mechanism to control and regulate the manufacture, supply, promotion, marketing, advertising, distribution and use of drugs, poisons, chemicals, cosmetics, medical devices, and food for human or animal use.*

The legislation goes on to define “medical device” and states that it is necessary to apply for authorization for all medical products including devices but does not provide any further guidance on the registration process.

#### 2. Regulatory Bodies

Regulatory authorities provide initial infrastructure to implement medical device law and prioritize the inclusion of national regulatory strategies. With the exception of Mozambique, all COSECSA member countries and South Africa have established national medicines regulatory authorities responsible for regulating medical devices.^3^ Mozambique uses the Pharmaceutical Department within its Ministry of Health as its regulatory authority but only mandates the regulation of drugs, not devices.^23^ The practical enforcement capacity of country regulatory authorities remains limited, particularly within Level 2 and Level 3 designated countries. Botswana's Medicines Regulatory Authority, for example, only has regulatory procedures in place for drugs and related substances but not devices.^30^

The practical enforcement capacity of country regulatory authorities varies and remains limited, particularly within Level 2 and Level 3 designated countries.

#### 3. Risk-Based Device Classification System

The most well-established regulatory systems classify devices according to risk. Medical devices vary in level of invasiveness, duration of use, and other technical elements that necessitate they be regulated according to stringent controls.^11^ A stethoscope, for example, poses a significantly lower risk to patients than a pacemaker. An understanding of the internationally harmonized risk-based classification system is necessary for governments seeking to develop regulatory strategies and for manufacturers seeking to enter markets in this region.

South Africa and most Level 2 designated countries use risk-based classification systems. South Africa,^24^ Kenya,^31^ Sudan,^32^ Tanzania,^33^ and Ethiopia^34^ employ a system that designates 4 levels of risk. South Africa is the only country included in this analysis that includes specific guidelines governing the regulation of in vitro diagnostic devices.^35^

#### 4. Essential Principles and Standards

A legal framework for regulatory processes should require that device manufacturers and importers present evidence of conformity to safety and performance standards. The IMDRF established a list of essential principles for medical devices including in vitro diagnostic devices.^11^ These principles included: (1) design and production processes should ensure that a medical device when used according to the intended purpose is safe and does not compromise the clinical condition of the patient or the health of the user; (2) the manufacturer should perform a risk assessment to identify known and foreseeable risks and to mitigate these risks in the design, production and use of the medical device; (3) under normal conditions, devices should perform as intended by the manufacturer; (4) performance and safety should not be affected during the lifetime of a medical device in a way that affects the safety of the user or patient; (5) performance and safety should not be affected by transport, packaging and storage; and (6) known and foreseeable risks should be weighed against the benefits of the intended purpose.

The regulatory processes for medical devices in South Africa^24^ and a number of Level 2 designated countries including Ethiopia,^34^ Kenya,^36^ Sudan,^32^ and Tanzania^33^ mandate conformity to these principles or an adaption within their guiding regulatory legislation. Ethiopia, for example, includes medical device essential safety and performance requirements within the *Guideline for Registration of Medical Devices*.^37^

#### 5. Conformity Assessment

The WHO maintains that the legal framework for medical devices should include a requirement that organizations seeking to market a medical device within the jurisdiction of a national regulatory authority must submit a declaration of conformity. A declaration of conformity corroborates that the device complies with the law or with certain accredited international standards. This should include a device description, adherence to a quality management system, and the presentation of technical documentation of safety and performance testing.^11^

Conformity assessment requirements vary among South Africa and Level 2 designated countries including Kenya,^31^ Sudan,^32^ Tanzania,^33^ and Uganda.^38^ Uganda, for example, requires that medical devices not licensed in 1 of the 5 IMDRF founding members (United States, European Union, Canada, Japan, or Australia) demonstrate conformity to WHO guidelines or to a quality management system used in IMDRF countries.^39^ Zimbabwe^28^ is the only Level 3 country that requires conformity assessment but does so only for gloves and condoms and not all medical devices.^28^ The Medicines Control Authority of Zimbabwe performs control assessments of gloves and male condoms in accordance with international standards and WHO guidelines due largely to their role in preventing the transmission of HIV/AIDS.

#### 6. Required Registration and Listing

There must be effective oversight of medical devices and those organizations responsible for bringing those devices to market. This is particularly relevant to COSECSA countries as many rely almost entirely upon imported medical devices.^8^ Many countries require devices, manufacturers, importers, and distributors to be registered with the national medicines regulatory authorities. This provides a greater potential for monitoring and postmarket inspection of medical devices to maintain adherence to quality standards over time. Registration and listing are required by all Level 1 and Level 2 countries. Zimbabwe requires registration and listing but only for parties who sell condoms or gloves as mentioned previously.^28^

#### 7. Import Controls

Imported medical devices must be approved before their shipment and entry. These controls provide regulators with advanced notice to verify if these devices have been previously marketed in the country and whether they conform to regulatory standards. Import controls are especially important in countries where most medical devices are imported. In South Africa, for instance, imported medical devices make up an estimated 90% of the market.^40^ South Africa and the majority of Level 2 countries have import controls. Rwanda and Zimbabwe are the only Level 3 countries with import controls.^26^^,^^28^

#### 8. Postmarket Controls

Regulatory authorities must address problems with registered medical devices as they arise. Medical devices do not always perform as expected, and there must be mechanisms to manage problems in design, manufacturing, performance, labeling, storage, distribution, or use.^41^ Controls can include a system for reporting complaints, inspection, procedures to withdraw from the market medical devices deemed unsafe, and market surveillance.^11^

Medical devices don't always perform as expected. Postmarket controls can help identify and manage problems in design, manufacturing, performance, labeling, storage, distribution, or use.

South Africa employs extensive postmarket controls including inspection per quality management systems procedures and guidelines, the seizure of devices that are unregistered or expired, reporting of adverse events, and controls of labeling and advertising.^42^ All Level 2 countries (Kenya, Ethiopia, Sudan, Tanzania, Uganda, and Zambia) likewise have postmarket controls in place, to varying degrees.^31^^–^^34^^,^^38^^,^^43^ Zambia, for example, has controls in place for the inspection, advertising, and labeling of devices but does not have a formal avenue for reporting adverse events. Rwanda is the only Level 3 country that employs postmarket control for all medical devices, but they are restricted to inspection, advertising, and labeling.^26^ However, inspection operates under the same guiding principles as all pharmaceuticals and food.

### Case Studies in Categorization Levels 1, 2, and 3

To gain a greater understanding of the unique regulatory processes and categorization schemes within East, Central, and Southern Africa, 3 countries (1 from each level) and their regulatory processes are reviewed in depth below.

#### Level 1: South Africa

In South Africa, medical devices are regulated by the South African Health Products Regulatory Authority under the *Medicines and Related Substances Act of 2015, Act No.1417*;^44^ *General Regulations Relating to Medical Devices and In Vitro Diagnostic Medical Devices*;^45^ and *Hazardous Substances Act No. 15 of 1973*.^24^ Specific guidelines for medical device standards are outlined in *General Information on Medical Devices and IVDs and Medical Devices and IVDs Essential Principles*.^46^

South Africa uses a risk-based classification system ranging from Class A (low risk) to Class D (high risk) to determine the premarket approval process. All pathways require appointing an authorized representative in South Africa. For Class A, devices demonstrate conformity by passing a Conformity Assessment Body and Declaration of Conformity. For Classes B-D, devices are required to meet the *Essential Principles* and demonstrate conformity by passing a Conformity Assessment Body and Declaration of Conformity. Passing the conformity assessment may require clinical testing, ensuring risk management, and outlining provisions for quality assurance techniques and sterility.^35^ Lastly, all medical devices, except custom-made devices, must be registered with the South African Health Products Regulatory Authority. All importers and manufacturers importing or exporting medical devices must also obtain a license from South African Health Products Regulatory Authority.^46^

Postmarket controls include inspections and certification of a quality management system. If medical devices fail to comply with postmarket requirements or are not registered, they can be seized under *General Regulations Relating to Medical Devices and In Vitro Diagnostic Medical Devices, Art. 16*.^35^ Advertising is permitted for certain audiences, such as health professionals. All medical device labels are in English. Applicants or holders of a device registration certificate are obligated to report detrimental effects associated with that device. Effective postmarket surveillance will require an avenue for consumers, providers, and distributors to report this information, and for the information to reach the device manufacturer. The institution and operationalization of this kind of reporting system will demand high enforcement capacity.

#### Level 2: Uganda

Within Uganda, the National Drug Authority (NDA) regulates medical devices according to the mandate presented in the *National Drug Policy and Authority Act, Cap. 206*.^47^ Standards and regulatory procedures including the definition of medical devices are outlined in the *Guideline for Registration of Medical Devices for Human Use In Uganda*.^39^ All medical devices manufactured, imported, and distributed in Uganda must be registered with the NDA. This excludes devices for which specific guidelines exist, namely malaria rapid diagnostic tests. In addition, the Uganda National Bureau of Standards, under the Ministry of Trade, formulates and enforces the use of standards.^48^

Registration does not require devices to be classified according to a risk-based system, but the NDA does offer 3 tracks that vary in complexity. Track 1 applications are reserved for devices already licensed in IMDRF countries and require less rigorous documentation. Track 2 applications are used for devices that are not licensed by IMDRF member countries. They can demonstrate evidence of conformity to a quality system standard from a certification body in 1 of the IMDRF founding member countries, WHO Prequalification, or other international organizations recognized by NDA. Lastly, Track 3 applications are required for devices that do not have certification of compliance to quality system standards. These applications require a Declaration of Conformity to IMDRF Essential Principles of Safety and Performance and information regarding preclinical design verification and validation. Maintenance of registration is reliant upon consistent quality, satisfactory performance of the device, and a 5-yearly registration review process. The NDA performs physical inspection of locally manufactured medical diagnostics annually. Imported devices are subject to inspection by the NDA at the port of entry.

In total, Uganda's process for the regulation of medical devices includes most components detailed in the Table, but the practical implementation of regulations remains limited. Efforts to control the safety and efficacy of imported medical devices prioritize malaria, HIV, and tuberculosis control programs.^8^ These disease areas remain at the apex of the Western global health agenda and tend to receive significant levels of global health assistance funding.

#### Level 3: Botswana

Botswana's National Regulatory Authority for medical devices is the Botswana Medicines Regulatory Authority, which was established under the *Medicines and Related Substances Act of 2013*.^44^ However, this legislation is still general and has not translated to the creation of formal avenues for device regulation. Botswana does not have a formal premarket approval process or postmarket surveillance. The Botswana Medicines Regulatory Authority is primarily focused on working toward implementing quality management systems to oversee the use of medical devices.^25^ Botswana does not have formal import regulations.

## DISCUSSION

### Availability of Literature

Peer-reviewed literature relating to regulatory processes for medical devices in Africa is very limited. This stands in stark contrast to the body of research around the FDA (U.S. Food and Drug Administration) approval process and the CE mark (European conformity mark). A simple literature search revealed 6,138 articles related to the regulatory process in Africa (of which most were not relevant). In contrast, 1.3 million articles related to the FDA process and 2.5 million articles related to the CE mark appear in a simple literature search. Considering this dearth in the literature, increased efforts should be directed toward developing the regulatory processes of African nations.

### Inadequate Regulatory Capacity and Enforcement

The majority of COSECSA member countries currently do not effectively regulate medical devices, due in part to both underdeveloped regulatory frameworks and a lack of downstream enforcement. Failure to successfully implement basic controls for the regulation of medical devices poses serious challenges for countries who wish to pursue more expanded controls and harmonize to international standards.^43^ Numerous political and socioeconomic conditions have restricted the ability of countries within East, Central, and Southern Africa to pursue the effective regulation of medical devices.^14^^,^^44^^,^^45^ As it stands, there are conflicting recommended approaches to build state capability and subsequently expand the capacity of COSECSA member countries to regulate the marketing of medical devices. These include the institutional approach and the problem-driven approach.^11^

Numerous political and socioeconomic conditions have restricted the ability of countries within East, Central, and Southern Africa to pursue the effective regulation of medical devices.

The institutional approach has largely been the preferred approach of major international bodies, such as the World Bank Group and the World Trade Organization.^49^ The institutional approach encourages the implementation of “best practices” with a focus on improving regulatory capacity. In theory, this empowers countries to expand regulatory capacity in a way that is sustainable, enforceable, and responsive to national public health priorities and resource availability.^20^^,^^50^^,^^51^ On the other hand, critics of this approach have raised concerns about its efficacy, especially in terms of what Andrews call “isomorphic mimicry.” By trying to implement “best practices,” the institutional approach could discourage experimentation and the prioritization of country-specific issues.^49^ The presence of regulatory processes that resemble those of IMDRF member states may in actuality mask the inability of institutions within many countries in the region to effectively carry out any regulatory processes.

The problem-driven approach diverges from the institutional approach by prioritizing country-specific issues and enforcement over the blanket implementation of “best practices.” This approach allows for feedback loops and greater policy experimentation as issues arise.^49^

In considering the potential for strengthened regulatory systems to expand access to quality medical devices in East, Central, and Southern Africa, it is salient to also understand that many states within the region currently lack the capacity to effectively carry out these reforms. As Andrews et al. wrote^52^:

*… articulating a reasonable policy is one thing: actually implementing it successfully is another.*

Significant effort must be directed toward the practical implementation of the critical components of these regulatory frameworks.

### The Effects of Colonialism and Economic Status

From 1881 to 1914, several European nations formed colonies in Africa that made a lasting imprint on the development of these countries.^51^ This is reflected in the correlation found between the date of independence and the status of regulatory processes for medical devices. The legacy of colonialism has persisted despite the majority of COSECSA countries gaining independence in the 1960s.^20^^,^^51^ Arbitrary postcolonial borders negotiated by European powers failed to consider competing ethnic groups within newly formed states, which resulted in instability as a result of civil conflict and separatist movements.^20^ The First and Second Sudanese Civil Wars, for example, were waged for nearly 40 years, and resulted in the eventual formation of an independent South Sudan in 2011, which continues to be plagued by civil conflict.^50^ South Sudan currently has no formal regulatory process in place for medical devices.

Additionally, a history of economic exploitation has manifested in the form of economic inequality, poverty, and class polarization. The instability within African nations, particularly those who have become independent in more recent years, has produced conditions which reduce the effectiveness of governance structures.^53^ Country governments may prioritize other health goals including poverty alleviation, the expansion of access to health care and the reduction of communicable diseases, which may be viewed as less consequential to the health and well-being of citizens.^54^

Country governments may prioritize other health goals over the medical device regulation, which may be viewed as less consequential to the health and well-being of citizens.

GDP and GDP per capita are both measures of economic conditions. GDP showed a strong positive correlation with the development of regulatory processes for medical devices, but GDP per capita was not as strongly correlated. GDP per capita is often used as a measure of prosperity and income inequality.^55^ The lack of correlation of GDP per capita with the development of regulatory processes could exist because GDP per capita is more telling of individual wealth. The total wealth available within a country is more accurately measured by the GDP and represents the resources that are available for community-wide investment, such as medical device regulation.

Innovative medical devices are needed to address the burden of disease, economic challenges, and infrastructure of African nations rather than just using medical devices that were designed for the needs and resources of high-income countries. Those seeking to develop such devices, both within and outside of Africa, face many challenges including clearing the regulatory processes of several countries and developing business models that provide sustainability.^56^

Many grants and awards for medical device innovations in Africa do not pay close attention to adherence to regulations—maybe due to their absence—but it leads many innovators to not value the importance of regulations in the early stages. Medical device regulation is not only needed to ensure patient safety but also to provide clarity, direction, and industry protection especially when substantial resources are invested into the development of a device. However, cumbersome regulatory processes and the risk of uncertain markets may prohibit medical device companies from developing technology suited to these regions.^57^

In many cases, innovation may stagnate because poor regulations and other factors result in an unreliable business environment. Better regulations around intellectual property protection would encourage local innovators as well as international business people to invest in the field.^17^ If African nations were to come together to develop a unified regulatory process, this would allow for pooling of resources, and relieve the economic and infrastructural burdens on individual countries.^58^ It would also simplify the process for device companies seeking to enter African markets, and therefore encourage innovation and provide an attractive market. Some efforts have been made to harmonize the regulatory process in Africa, but this has focused heavily on medications and less so on medical devices.^15^ There are many fragmented systems in Africa, representing large challenges. Investment in harmonization may be an opportunity to provide synergy for other fragmented systems to grow together.

One could argue that African nations could just accept the CE Mark or FDA approval, which is effectively what many countries are currently doing. However, this is not ideal as the FDA and CE mark processes were designed for the needs of high-income countries. The review process may not consider infrastructural limitations currently present in many African nations. Many medical devices designed to meet the standards of other countries have been observed to easily malfunction due to such factors. In addition, cultural and economic barriers may prohibit African medical device companies from obtaining approval through these entities.

### Limitations and Complexities

Although an understanding of the extent to which COSECSA member countries have a regulatory framework is valuable, it is crucial to recognize that the mere presence of a regulatory framework for medical devices does not predict more effective government oversight of the provision of health-related goods and services. Likewise, classification as Level 3 does not inherently mean that a country has similarly weak health infrastructure. The stringency of required regulatory processes may serve as a helpful proxy for the efficacy of government measures for the oversight of health-related goods and services, but it is not a steadfast rule. For example, although we classified Botswana as Level 3, it has a significantly more robust health care system than many other COSECSA member states.

Despite a long colonial history, an independent Botswana has achieved what many scholars call an “exceptional” record of functioning institutions and consistently favorable economic growth.^59^ The breadth of health services provision in Botswana is consistent with this claim. The nation relies on integrated primary health care as the basis of the health delivery system. The Ministry of Health is predominantly responsible for national health policy and strategies for health development and delivery whereas 27 decentralized health districts are responsible for the provision of public sector services. Public health services which are nearly free for citizens emphasize preventative over curative medicine and have allowed for steady increases in equitable access to health care for the citizens of Botswana.^60^ Additionally, the U.S. International Trade Administration reports that in recent years, Botswana's government has prioritized human resources development, technology, and supply chain capacity.^61^ These factors in combination with a higher national GDP and a national policy on health technology demonstrate that the government of Botswana may be more likely to ensure access to quality medical devices than some COSECSA counterparts despite the country's lack of a formal regulatory framework.

Cases like Botswana indicate that the mechanism for expanding access to medical devices is more complex than the sole existence of a regulatory framework. Future work must also consider the interplay between regulatory frameworks, political, and socioeconomic institutions and existing health infrastructure.

## RECOMMENDATIONS

With these challenges in mind, several strategies may be pursued to build state capability and mitigate the economic and health effects of weak regulatory systems for medical devices. We propose recommendations that may prove useful to policy makers and other stakeholders to improve regulatory systems within COSECSA member countries and beyond.

Policy makers may choose to employ an institutional approach toward expanding regulatory capacity by adopting and amending existing harmonized regulations, rather than formulating new ones. The IMDRF facilitates the adoption of regulations for medical devices developed by member countries. It grants low- and middle-income countries the opportunity to participate in and observe well-established regulatory development processes and take advantage of recent medical advancements. Therefore, it is advisable for low- and middle-income countries to use this platform to minimize resource expenditure during the development and formulation of new regulations.

This strategy may, however, fail to consider country-specific barriers to enforcement. Thus, it is crucial to also prioritize local capacity building. Local capacity in the form of well-trained personnel, tools, and facilities is an essential driver of efficient and effective medical technology regulatory bodies in all countries. Locally trained, capable professionals with sufficient financial and technical support will be more capable of responding with authority to distinctively local challenges.

Capacity building requires resources. As noncommunicable diseases are increasingly recognized as a major source of morbidity and mortality globally, medical devices that address these issues must be prioritized. Grant funding from government and nongovernmental agencies should be used to promote innovation and capacity building in medical device regulation and harmonization. Specifically, many medical technology companies (primarily based in high-income countries) have a charitable arm and should consider investment in efforts that seed and encourage Pan-African harmonization. Currently existing African medical and engineering societies are instrumental in connecting professionals across country borders and should play an increased role in galvanizing these changes.

## CONCLUSION

The current landscape for regulation of medical devices within East, Central, and Southern Africa is complex and often underdeveloped, despite a legal mandate for regulation in most countries. Higher GDP and years of freedom from colonization were positively correlated with a country's regulatory capacity. A streamlined regulatory process, harmonized across African nations would simplify the regulatory process for companies and possibly make it less expensive and more efficient to bring medical devices to the African market, thereby increasing patient and physician access to medical devices and improving health outcomes.

## Notes

**Peer Reviewed**

**First published online:** March 25, 2021.
